# What Predicts Changes in the Work Situation of Recently Diagnosed People with Multiple Sclerosis and do these Predictors also Apply to Healthy People?

**DOI:** 10.1007/s10926-025-10279-2

**Published:** 2025-02-27

**Authors:** Shalina R. D. Saddal, Karin van der Hiele, Ehsan Motazedi, Elianne E. A. van Egmond, Leo H. Visser, Pauline T. Waskowiak, Amber van der Kruit, Maureen van Dam, Menno M. Schoonheim, Vincent de Groot, Hanneke E. Hulst, Frederieke G. Schaafsma

**Affiliations:** 1 MS Center Amsterdam, Public and Occupational Health, Vrije Universiteit Amsterdam, Amsterdam UMC Location VUmc, Van Der Boechorstraat 7, 1081BT Amsterdam, The Netherlands; 2 Health, Medical and Neuropsychology Unit, Institute of Psychology, Faculty of Social Sciences, Leiden University, Leiden, The Netherlands; 3 MS Center Amsterdam, Anatomy and Neurosciences, Vrije Universiteit Amsterdam, Amsterdam Neuroscience, Amsterdam UMC Location Vumc, Amsterdam, The Netherlands; 4 MS Center Amsterdam, Rehabilitation Medicine, Vrije Universiteit Amsterdam, Amsterdam UMC Location Vumc, Amsterdam, The Netherlands; 5 MS Center Amsterdam, Medical Psychology, Vrije Universiteit Amsterdam, Amsterdam Neuroscience, Amsterdam UMC Location Vumc, Amsterdam, The Netherlands; 6 Department of Public and Occupational Health, Amsterdam UMC, Universiteit Van Amsterdam, Amsterdam, The Netherlands; 7 Department of Neurology, Elisabeth-TweeSteden Hospital, Tilburg, The Netherlands; 8 University of Humanistic Studies, Utrecht, The Netherlands

**Keywords:** Multiple sclerosis, Work, Cognition, Conscientiousness

## Abstract

**Purpose:**

To study associations of baseline predictors with work difficulties and number of working hours after one year in recently diagnosed people with multiple sclerosis (PwMS). Furthermore, to analyze whether these predictors are generic, i.e., apply to healthy people as well, or are disease specific.

**Methods:**

TEMPRANO and MS@Work data were combined resulting in a dataset of 135 recently diagnosed PwMS (≤ 1 year) and 172 healthy people. We analyzed the associations of baseline predictors that fit within the international classification of functioning and health (ICF) framework using a mixed-effect negative-binomial model with log link for work difficulties and number of working hours after one year, and fitted each model using a fully Bayesian approach.

**Results:**

Slower information processing speed was a disease-specific predictor for more work difficulties after one year (posterior mean ratio (PMR) = 0.82, 95% confidence interval (CI) = [0.69, 0.97]). Higher conscientiousness was a generic predictor for more working hours after one year (PMR = 1.02, 95%CI = [1.01, 1.03] for PwMS and PMR = 1.01, 95% CI = [1.00, 1.02] for healthy people). Being male (PMR = 0.75, 95% CI = [0.58, 0.96]), being younger (PMR = 0.99, 95% CI = [0.98, 1.00]), higher information processing speed (PMR = 1.08, 95% CI = [1.04, 1.13]), better learning and memory (PMR = 1.09, 95% CI = [1.03, 1.15]), and mentally burdensome job tasks (PMR = 1.36, 95% CI = [1.22, 1.52]) were disease-specific predictors for more working hours after one year.

**Conclusion:**

For recently diagnosed PwMS, disease-specific predictors that fit within the ICF framework associate with perceived work difficulties and working hours after one year.

**Supplementary Information:**

The online version contains supplementary material available at 10.1007/s10926-025-10279-2.

## Introduction

Multiple sclerosis (MS) is the most prevalent neurological disorder affecting young and middle-aged adults [1] and worldwide the number of MS cases is increasing [2]. The disease is commonly diagnosed in adults of working age ranging from 20 to 49 years old [3]. Although adults in this age range are generally in the prime of their careers, the reported unemployment rates in people with MS (PwMS) are up to 80% [4]. Interestingly, most PwMS are employed before their diagnosis [5] and nearly half appear to leave their job within the first 3 years after diagnosis [6]. This early period following diagnosis may thus be a crucial period for interventions that aim to prevent early unemployment in PwMS.

Unfortunately, studies investigating predictors for early unemployment in recently diagnosed PwMS are scarce, as most studies have included PwMS with an average disease duration of 9 to 14 years [7]. Moreover, these studies included combinations of a relatively small number of disease-related predictors such as physical and cognitive functioning [8, 9]. Although different studies investigated other predictive factors (such as personal or environmental factors) related to unemployment in PwMS as well, and found a wide variation of factors to be associated with unemployment in this population [10], there are no studies that investigated a combination of different factors in recently diagnosed PwMS. The importance of studying a combination of different predictive factors is further emphasized by the International Classification of Functioning, Disability and Health (ICF), a global conceptual framework of functioning [11]. As depicted in the ICF framework, a combination of body functions, personal factors, and external factors may influence activities and participation (e.g., employment) in relation to a disease (e.g., MS). Since unemployment in PwMS starts soon after diagnosis, research focusing on factors at these different levels within the first months after diagnosis is of the essence.

Multiple studies found associations with different predictors within three components of the ICF framework. Strober (2020) prospectively studied determinants of unemployment in PwMS and found body functions (fatigue, sleep, and pain), personal factors (personality, coping, and self-efficacy), and engagement in positive health-related behaviors to be associated with the risk of becoming unemployed [4]. Worse disease course and longer disease duration, lower level of education, and higher age were found to characterize unemployed PwMS [7]. Other personal factors such as effective coping and better physical and/or cognitive functioning were associated with more favorable outcomes of employment in PwMS [12–14]. Work-related factors, i.e., nature of the work tasks and perceived work difficulties, were also found to be related to employment in PwMS [15].

Because employment is a multidimensional concept, an integrative approach including factors from multiple components of the ICF framework in recently diagnosed pwMS is needed. Also, although these studies give an indication of which predictive factors may be prognostic for employment in PwMS none included a healthy control group. Consequently, it is impossible to conclude whether these disease-specific predictors for employment also play a role in employment in a healthy population. In addition, investigating differences between PwMS and healthy people will provide insight into possible relevant factors for changes in work difficulties and number of working hours that allow us to make more substantiated recommendations for intervention strategies to prevent early unemployment in PwMS in particular.

In this study, we aim to identify body functions, and personal and external factors that are associated with work difficulties and number of working hours after one year in recently diagnosed PwMS, as early unemployment in PwMS is often preceded by experiencing more work difficulties and a decrease in the number of working hours[16, 17]. In addition, we aim to study to what extent these predictive factors are generic, i.e., apply to a healthy population as well, or are disease specific. Based on the literature, we expect that a combination of factors that fit across three components of the ICF framework is associated with work difficulties and working hours in recently diagnosed PwMS.

## Methods

### Design

In this study, we examined whether a combination of body functions, personal factors, and external factors can predict work difficulties and number of working hours after one year in recently diagnosed pwMS, as well as in healthy people. Data from two different studies were combined to create a sufficient sample size (N = 287): (1) Temprano is a 2-year prospective, single-center, observational cohort study. Data collection is ongoing in recently diagnosed PwMS (6–12 months after diagnosis) and matched healthy people. Temprano was approved by the Medical Ethics Committee of Amsterdam UMC (Register CCMO: NL72064.029.20) and all included participants signed an informed consent form, (2) The MS@Work study is a multicenter 3-year prospective observational study in PwMS and healthy people[18]. Only study participants with a diagnosis duration up to 12 months at baseline were included in this study. The MS@Work study was approved by the Medical Ethical Committee Brabant (Register CCMO: NL43098.008.12 1307) and included participants signed an informed consent form. A list of study parameters of both studies can be found in Supplementary materials 1.

### Study Population

Our sample consisted of 135 people who, at the time of the baseline measure, were diagnosed with MS up to 12 months ago according to the McDonald 2017 criteria[19] and 172 healthy people. All participants were aged between 18 and 67 years old.

Additional inclusion criteria for both studies were sufficient Dutch language proficiency and for PwMS a diagnosis with a relapsing remitting disease course. Participants were excluded if they had (a history of) psychiatric or neurological disorders or substance abuse. Participants in the Temprano study had to meet standard safety criteria to undergo MRI examination and were excluded if they have had relapses or steroid treatments less than four weeks prior to the visit. Participants in the MS@Work study had to be either employed or within three years since their last employment at the moment of inclusion in the study and healthy people were excluded on having chronic disorders of any kind. Study visits were rescheduled in case of an MS relapse within 1 month prior to the study visit.

### Outcome Variables

Work difficulties after one year were assessed using the total score on the MS Work Difficulties Questionnaire-23[20] (MSWDQ), in both the MS population and the healthy control group. In this questionnaire, work difficulties (which mainly occur in PwMS) were assessed using 23 items across three broad domains: psychological/ cognitive barriers, physical barriers, and external barriers. Participants indicated how often they experienced certain situations on a five-point Likert scale ranging from “never” to “almost always” on items such as “I felt that I had to work because of my financial position” and “I felt socially isolated.” Scores on the MSWDQ ranged from 0 to 100, with higher scores indicating the presence of more work difficulties.

We also looked into the total number of self-reported working hours per week after one year.

### Baseline Predictors

We selected baseline predictors from overlapping variables measured at baseline in the Temprano and MS@Work datasets, using the ICF framework[11] as guidance.

We categorized these predictors into body functions, personal factors, and external factors.

### Body Functions

For body functions, we selected four variables: use of disease modifying therapies, fatigue, and two domains of cognitive functioning.

- The use of disease modifying therapies (DMT’s): for the purpose of analysis, we distinguished between the use of none or first-line compared to second-line medications. This distinction is also informative about disease activity, as second-line medications are considered when first-line medication is ineffective or the disease progresses[21].

- Scores on the sub-scale ‘subjective fatigue’ of the Checklist of Individual Strength (CIS-20) represents subjective fatigue and fatigue-related behavioral aspects[22]. The complete Dutch questionnaire consists of 20 items and 4 sub-scales: subjective fatigue, concentration, motivation, and physical activity. Participants have to indicate on a Likert scale from 1 to 7 ranging from “yes that is true” to 7 “no that is not true” whether they agree with statements such as “I feel tired” and “I don’t do much during the day.” Scores on the subjective-fatigue sub-scale range from 8 to 56 and higher scores indicate the presence of more subjective fatigue.

- Two domains of cognitive functioning: information processing speed and learning & memory, as these are the most common cognitive impairments in PwMS[23]. Scores on the Paced Auditory Serial Addition Test[24] (PASAT) represent information processing speed. Scores on the learning and recall trials of the Dutch version of the California Verbal Learning Test[25] (CLVT-II) (in the Temprano dataset), Dutch version of the Auditory Verbal Learning Test[26] (AVLT) (in the MS@Work dataset), and Brief Visuospatial Memory Test-Revised[27] (BMVT-R) represent learning & memory. We calculated z-scores for each participant on both domains using regression-based norms, correcting for the effects of age, sex, and educational level, from a large dataset that was collected at the Amsterdam UMC, location VUmc, and Leiden University[28, 29]. Higher z-scores on each domain indicate better cognitive functioning in that specific domain.

### Personal Factors

For personal factors, we selected five variables: sex, age, level of education, personality, and coping. In selecting variables as part of this component, we were limited to selecting a maximum of two extra predictors in addition to the demographical variables to ensure sufficient statistical power in the analyses. We decided to favor personality and coping as predictors over depression and anxiety. Although there is evidence for associations between all these factors and employment outcomes in MS[14, 30, 31], we chose emotion-focused coping as this promising and potentially modifiable factor has not yet been extensively studied in pwMS in relation to employment[14]. The personality trait ‘conscientiousness’ has recently been identified as an interesting modifiable predictor of work outcomes in MS[31].

- Sex (male/female), age (in years), and level of education (Dutch school system) were added as predictors for personal factors to the model. Level of education was classified using the Verhage classification[32] and was categorized as predominantly practically educated (completed low-level secondary school), or predominantly theoretically educated (completed secondary school at medium or the highest level) for the purpose of analyses.

- The personality trait ‘conscientiousness’ was measured using the NEO Five-Factor Inventory (NEO-FFI [33]). This 60-item questionnaire contains 12 items concerning conscientiousness. Participants have to indicate on a five-point Likert scale ranging from “strongly disagree” to “strongly agree” to which extent they agree with each item. Examples of items concerning conscientiousness are “has a clear set of goals” and “follows through on commitments”[34]. Scores on the conscientiousness sub-scale range from zero to 60 and a higher score indicates a higher level of conscientiousness.

- Coping: coping was not measured with the same questionnaire in the two studies from where the data were collected. A study on the convergent validity of the Utrechtse Coping Lijst (UCL[35]) and the Coping Inventory for Stressful Situations (CISS[36]) found a strong coherence between the sub-scale “emotion-focused coping” from the CISS and “passive reaction pattern” from the UCL[37]. We dichotomized the coping variable using only the strongly coherent sub-scales from the CISS and UCL. Because the distribution of scores was skewed, the median was used as cutoff score for categorizing scores as either low emotion-focused coping style (1) or high emotion-focused coping style (2).

### External Factors

For external factors, we only considered the dominant nature of work tasks (physically vs mentally burdensome) as a potential predictor. Participants indicated whether they considered the tasks they perform at work “physically burdensome,” “mentally burdensome,” or “both physically & mentally burdensome.” For the purpose of analyses, we dichotomized this variable in “mentally burdensome” and “physically burdensome.” Participants who answered “both physically & mentally burdensome” were added to the “physically burdensome” category since not every job has obvious physical demands (while mentally straining tasks occur in most occupations nowadays due to developments such as digitalization and automation processes[38]).

### Statistical Analyses

We investigated associations of potential prognostic variables measured at baseline, with MSWDQ scores and number of working hours measured after one year in the healthy population and in the MS population. We performed multivariable analyses and compared the effects of each prognostic factor between the two populations. As the population sizes were small, we used a fully Bayesian approach to borrow information from the larger healthy population in the estimation of the effects in the MS population, with the aim of increasing the precision and statistical power compared to a simple stratified analysis. The models were corrected for baseline MSWDQ and working hours. Additionally, we kept all variables in the models to correct for confounding. Using this approach allowed us to get as close as possible to causal inferences.

We used a mixed-effect negative-binomial model with the log link for work difficulties and working hours after one year, and conducted posterior inference using Hamiltonian Monte Carlo (HMC) sampling implemented in Stan[39]. All models were also adjusted for the random effect of the data source, i.e., Temprano or MS@Work study.

The prior distribution of all predictors, i.e., fixed effects, in the healthy population was set to a weakly informative flat normal distribution. The prior distribution of each fixed effect in the MS population was set to a normal distribution with the mean and standard deviation obtained from the posterior mean and posterior standard deviation of the corresponding effect in the healthy population, except for the general intercept and the MS-specific prognostic factors, i.e., DMT, for which flat normal priors were used. In both populations, a weakly informative gamma prior was considered for the dispersion adjustment parameter of the negative binomial (φ ~ γ(0.5,0.1)), while a normal prior was considered for the random effect of the data source, having a zero mean and a standard deviation with a weakly informative half-Cauchy prior $\left( {\mu_{a} \sim N\left( {0, \sigma_{a} } \right), \sigma_{a} \sim Half - Cauchy\left( {0, 2.5} \right)} \right)$.

For each model, the posterior mean and 95% equal-tail credibility intervals were obtained for each predictor effect, from 6 posterior HMC chains with 5,000 warm-up and 10,000 post warm-up draws and a thinning factor of 2. The convergence of the chains was checked using the Brooks and Gelman potential scale reduction factor (split-R ^) [40], and the sampling efficiency was checked using the effective sample size (N_eff) [39].

Finally, the initial mean value of the dependent variable and the posterior mean ratio for significant associations were used to calculate the new mean value and the absolute change. The absolute change was calculated as the difference between the new mean value and the initial mean value. To provide a better understanding of the clinical relevance of significant associations, we presented the absolute change in the outcome variable per 10% change in the independent variable.

## Results

Descriptive characteristics of the complete study population are observed in Table 1. We performed complete case analyses excluding participants with missing values on one of the variables in the model. The actual sample sizes used in each model and descriptive statistics for these sub-populations are shown in Supplementary materials 2.

**Table 1:** Descriptive (baseline) characteristics of the complete study population

	HC		MS	
Variable	Total n	n (%)	Total n	n (%)
Sex*	169	123(72.8%) female	135	115(85.2%) female
Education*	169		134	
- Practical		42(24.9%)		58(43.3%)
- Theoretical		127(75.1%)		76(56.7%)
Disease modifying treatment			115	
- None or first line	–	–		97(84.3%)
- Second line	–	–		18(15.7%)
Emotion-focused coping	169		128	
- Low		91(53.8%)		67(52.3%)
- High		78(46.2%)		61(47.7%)
Work tasks*	162		99	
- Mentally burdensome		99(61.1%)		23(23.2%)
- Physically burdensome		63(38.9%)		76(76.8%)

		Mean (SD)		Mean (SD)
Age in years*	172	39.56(11.3)	132	36.87(9.53)
Fatigue*	169	23.08(10.96)	128	34.08(11.79)
Information processing speed (z-score)	172	− 0.09(1.00)	122	− 0.28(11.79)
Learning and memory (z-score)*	172	− 0.01(.57)	123	− 0.27(.83)
Conscientiousness*	172	47.07(5.88)	126	46.29(.491)
Work difficulties				
- Baseline*	169	9.22(9.81)	128	23.58(14.59)
- One-year follow-up*	99	7.99(9.30)	90	21.56(15.27)
Working hours per week				
- Baseline*	154	34.31(9.16)	87	29.21(10.46)
- One-year follow-up*	129	33.87(9.09)	73	28.71(10.32)

*= significant group difference between HC and pwMS at *p* < .05 assessed with chi-square test (sex, education, disease modifying treatment, emotion-focused coping, work tasks) and t-test (age, fatigue, information processing speed, learning and memory, conscientiousness, work difficulties, working hours per week)

*HC* healthy controls, *MS* recently diagnosed people with multiple sclerosis

Total n represents the n for each variable excluding missing. Education categories are combinations of Verhage[30] scores. Practical = scores 1–5, Theoretical = scores 6 and 7

Fatigue: range = 8–56, higher = more fatigued. Information processing speed (z-score)**:** higher = better. Learning and memory (z-score**):** higher = better

Conscientiousness: range = 27–60, higher = more conscientious. Work difficulties: higher = more work difficulties, baseline range = 0–67.05, One-year follow-up range = 0–63.64

Working hours per week: baseline range = 0–70, One-year follow-up range = 0–70

Significant group differences between HC and pwMS were assessed using chi-square tests and t -tests and are indicated in Table 1. In the healthy control group, a larger proportion of people have a predominantly theoretical education compared to a practical education. Moreover, a larger proportion of recently diagnosed PwMS reported their work tasks more physically burdensome (as opposed to mentally burdensome) at baseline. In addition, recently diagnosed PwMS scored higher on fatigue and scored worse on cognitive tests (measuring information processing speed and learning and memory) compared to healthy people. Finally, recently diagnosed PwMS experienced more work difficulties and worked on average less hours a week compared to healthy people at baseline as well as after one year. On average, both healthy people and PwMS scored approximately two points lower on the MSWDQ and worked one hour less after one year than at baseline.

### Work Difficulties after One Year

The posterior mean ratios and 95% equal-tail credible intervals (CI) of the regression coefficient in the mixed-effect negative-binomial model for work difficulties after one year are shown in Table 2, for the healthy population and the population with MS including 97 and 61 individuals, respectively**.**

**Table 2:** Associations of body functions, personal factors and external factors, with work difficulties and number of working hours after one year

	Work difficulties after one year Posterior mean ratio [95% CI]		Working hours after one year Posterior mean ratio [95% CI]	
	HC	MS	HC	MS
**Body functions**				
Disease modifying treatment (DMT)	–	0.87 [0.46, 1.67]	–	1.08 [0.78, 1.50]
Fatigue	1.02 [0.99, 1,05]	1.01 [0.99, 1.03]	1.00 [0.99, 1.00]	1.00 [1.00, 1.01]
Learning and memory	1.41 [0.88, 2.29]	1.02 [0.82, 1.26]	1.00 [0.93, 1.08]	1.09 [1.03, 1.15]*
Information processing speed	0.79 [0.63, 1.01]	0.82 [0.69, 0.97]*	0.97 [0.93, 1.02]	1.08 [1.04, 1.13]*
**Personal factors**				
Sex (female vs male)	1.21 [0.66, 2.24]	0.90 [0.50, 1.56]	1.01 [0.91, 1.10]	0.75 [0.58, 0.96]*
Age	0.99 [0.97, 1.02]	1.01 [0.98, 1.03]	0.99 [0.99, 1.00]	0.99 [0.98, 1.00]*
Education (theoretical vs practical)	0.93 [0.50, 1.72]	1.37 [0.93, 2.05]	0.95 [0.86, 1.06]	1.11 [0.97, 1.27]
Conscientiousness	0.97 [0.93, 1.02]	0.97 [0.94, 1,00]	1.01 [1.00, 1.02]*	1.02 [1.01, 1.03]*
Emotion-focused coping	1.20 [0.74, 1.94]	1.09 [0.78, 1.51]	0.99 [0.91, 1.08]	0.98 [0.90, 1.06]
**External factors**				
Work tasks (mentally vs physically burdensome)	1.15 [0.62, 2.09]	1.20 [0.80, 1.82]	1.07 [0.96, 1.18]	1.36 [1.22, 1.52]*
Work difficulties baseline	1.09 [1.05, 1.14]*	1.04 [1.03, 1.06]*	1.00 [1.00, 1.01]	1.00 [0.99, 1.00]
Working hours baseline	1.01 [0.99, 1.04]	1.01 [1.00, 1.03]	1.02 [1.02, 1.03]*	1.02 [1.02, 1.03]*

*HC* healthy controls, *MS* recently diagnosed people with multiple sclerosis

* = Significant at p < 0.05

#### Generic Associations (Applying to both HC and pwMS)

In the healthy population, the posterior mean ratio for work difficulties was 1.09 (95% CI = [1.05, 1.14]), corresponding to a 7.30 points higher work difficulties score after one year per 10% higher work difficulties score at baseline. In the MS population, the posterior mean ratio was 1.04 (95% CI = [1.03, 1.06]), corresponding to a 8.40 points higher work difficulties score after one year per 10% higher work difficulties score at baseline. These findings suggest that in general, more work difficulties at baseline are associated with more work difficulties after one year.

#### Disease-Specific Associations (Applying Solely to pwMS)

The posterior mean ratio for information processing speed was 0.82 (95% CI = [0.69, 0.97]), corresponding to a 2.27 points lower work difficulties score after one year per 10% higher information processing speed at baseline. This finding suggests that for PwMS, better information processing speed at baseline is associated with less work difficulties after one year.

### Working Hours after One Year

The posterior mean ratios and 95% equal-tail credible intervals of the regression coefficient in the mixed-effect negative-binomial model for working hours after one year are shown in Table 2 for the healthy population and the population with MS, including 129 and 52 individuals, respectively.

#### Generic Associations

In the healthy population, the posterior mean ratio for working hours was 1.02 (95% CI = [1.02, 1.03]), corresponding to 4.76 more working hours after one year per 10% more working hours at baseline. For PwMS, the posterior mean ratio for working hours was 1.02 (95% CI = [1.02–1.03]), corresponding to 3.99 more working hours after one year per 10% more working hours at baseline.

In the healthy population, the posterior mean ratio for conscientiousness was 1.01 (95% CI = [1.00, 1.02]), corresponding to 2.04 more working hours after one year per 10% higher conscientiousness score at baseline. For PwMS, the posterior mean ratio for conscientiousness was 1.02 (95% CI = [1.01–1.03]), corresponding to 3.42 more working hours after one year per 10% higher conscientiousness at baseline.

These results suggest that in general, higher conscientiousness and more working hours at baseline are associated with more working hours after one year. The posterior mean ratio and absolute change for conscientiousness are slightly larger in PwMS, indicating that conscientiousness may be particularly important in the early phase after MS diagnosis.

#### Disease-Specific Associations

The posterior mean ratio for learning and memory was 1.09 (95% CI = [1.03–1.15]), corresponding to 1.54 more working hours after one year per 10% higher learning and memory at baseline. Additionally, the posterior mean ratio for information processing speed was 1.08 (95% CI = [1.04–1.13]), corresponding to 1.37 more working hours after one year per 10% higher information processing speed at baseline. These results suggest that, for PwMS, better learning and memory and higher information processing speed at baseline are associated with more working hours after one year.

Males with MS worked 25% more hours after one year compared to females with MS (posterior mean ratio = 0.75, 95% CI = [0.58–0.96]), corresponding to an absolute change of 7.13 h. The posterior mean ratio for age was 0.99 (95% CI = [0.98–1.00]), corresponding to 1.42 less working hours after one year per 10% higher age at baseline. Finally, PwMS who consider their job tasks mostly mentally burdensome work 36% more hours after one year compared to PwMS who also consider their job physically burdensome (posterior mean ratio = 1.36, 95% CI = [1.22–1.52]). The absolute change was 10.26 h, indicating that PwMS who consider their job mostly mentally burdensome worked 10.26 more hours after one year compared to PwMS who also consider their job physically burdensome. These results indicate that being male, being younger, and having more mentally burdensome job tasks (as opposed to more physically burdensome job tasks) are associated with more working hours after one year.

## Discussion

In this study, we identified baseline predictors fitting the ICF framework that were associated with work difficulties and the number of working hours after one year in recently diagnosed people with MS (PwMS). In addition, we investigated whether these predictors differ for healthy people compared to recently diagnosed PwMS.

We found that PwMS who had a higher information processing speed score at baseline experienced less working difficulties after one year (10% higher information processing speed at baseline was associated with a 2.27 lower work difficulties score after one year). This finding confirms the results of earlier studies that found an association between information processing speed and early unemployment in PwMS [41]. The absence of a significant association in healthy people while it is present in recently diagnosed PwMS suggests that already within one year after MS diagnosis, the association between information processing speed and work difficulties is apparent.

We found that 10% higher conscientiousness at baseline was associated with a higher number of working hours after one year, in both healthy people (+ 2.04 h) and recently diagnosed PwMS (+ 3.42 h). Thus, conscientiousness may be an important general factor for job maintenance. The results of a large comprehensive review published in 2019 confirmed the importance of conscientiousness in employment in general [42]. More recently, this association has also been studied in PwMS specifically showing that reduced employment (fulltime to parttime) and negative work events in PwMS were associated with lower conscientiousness [31]. However, the MS@Work study—of which a selection of the data was also included in the current analysis—did not replicate these findings. In that study, the average disease duration among participants was five years, whereas in our study, only individuals with a disease duration of one year or less were included. This suggests that conscientiousness may play a particularly critical role in the earliest stages of MS. Additionally, the outcome measure in the current study—working hours after one year—enabled us to capture more subtle changes compared to the previous study’s broader outcomes, such as deterioration in employment status or an increase in negative work events (DES). It may be argued that a decline in working hours might fluctuate early after diagnosis and the average difference in working hours between baseline and after one year in the current study is very small. Nonetheless, we did find evidence for these changes in working hours to be related to disease-specific associations in this study. On top of that, fluctuations in working hours provide insight into the flexibility of the job (because it reflects whether it is possible to adapt working hours if needed) early after diagnosis [43]. This flexibility may affect job maintenance over time. Therefore, future studies investigating prognostic factors for employment in recently diagnosed PwMS should include number of working hours as outcome.

We found that PwMS who scored higher on learning and memory at baseline worked more hours after one year (10% higher learning and memory at baseline was associated with 1.54 more working hours after one year). Additionally, 10% higher information processing speed at baseline was associated with 1.37 more working hours after one year. These findings did not apply to the healthy population. However, PwMS already scored worse on these cognitive domains at baseline compared to the healthy population which indicates that differences in cognition may already take place very early after diagnosis or even before diagnosis. The effectiveness of intervening early on cognition and employment (before the presence of problems) is currently being studied [44]. In addition, our results suggest that training cognitive functions and conscientious behavior early after diagnosis may prevent a decrease in the number working hours in PwMS, which often precedes early unemployment [16]. Different studies found speed of processing training to be effective for improving processing speed in PwMS [45, 46]. Future studies may want to examine the direct effects of speed of processing training on sustaining employment, particularly early after diagnosis. Promising is the work of Fuchs et al. [47] in which they investigated an intervention targeting conscientiousness in PwMS and found positive effects on employment such as a work promotion and new work accommodations. Future interventions for sustaining employment early after diagnosis should also include effective elements from their studied intervention.

The results also showed that within the group of PwMS, males worked 7.13 more hours after one year compared to females. This may be explained by that women more often have the ‘secondary’ job of the household, while men are still typically seen as the primary earner. Earlier studies showed that being the main provider for the family is an important reason for people with chronic diseases to keep working [48]. However, we cannot be sure that is the case here since we do not have information in our dataset about whether PwMS are the main provider for their household. Future studies should include this question to further investigate the association between sex and employment in PwMS. Additionally, younger age in PwMS was associated with working more hours after one year. This corresponds with the idea that preventative interventions for early unemployment may be more effective earlier in the disease as younger PwMS are working more hours.

Finally, having mostly mentally burdensome job tasks at baseline was associated with 10.26 more working hours after one year compared to partly or mostly physically burdensome job tasks in PwMS. It is noteworthy that some PwMS reported their job tasks as (partly) physically burdensome, while these same job tasks were reported to be mentally burdensome by some healthy people (e.g., healthy people working as a teacher reported their job tasks to be more mentally burdensome, while PwMS working as a teacher reported their job tasks to be more physically burdensome). Thus, disease-specific factors may influence people’s perception of the type of burdensome work tasks. Moreover, at baseline, PwMS already reported experiencing their job tasks as more physically burdensome compared to healthy people. We were unable to include a representative proxy for physical functioning such as the expanded disability status scale score (EDSS) [49], which is unfortunate as earlier research did show that physical disability is an important predictor for employment status [50]. As more PwMS had a practical education (as opposed to a theoretical education) compared to healthy people, it may be the case in this study that more PwMS had a physically burdensome job. Alternatively, our finding may also imply that disease-related factors in the form of physical disabilities are already at play at work early after (or even before) diagnosis leading to more physical burden. Because better cognitive functioning was found to be associated with more working hours after one year, it might have been expected that more mentally burdensome job tasks associate with less working hours after one year. The results of the current study imply that both physical and cognitive functioning may be important factors for the number of working hours after one year in recently diagnosed PwMS. Therefore, future studies on employment in PwMS should aim to include both physical and cognitive factors.

We did not find any significant associations for DMT and work-related outcomes in the current study. A relatively small number of participants used second-line medication compared to none or first-line medication. Therefore, the predictive power of this variable may have been too low in the current study to identify any associations. Surprisingly, although PwMS scored higher on fatigue at baseline compared to healthy people, no significant associations were found for fatigue in the current study. It should be noted that in the current dataset, fatigue was strongly correlated with work difficulties and number of working hours at baseline. In the statistical analyses to answer the research questions of the current study, we predicted work difficulties and working hours after one year and corrected for baseline values. It could be that the absence of significant effects of fatigue on our work-related outcome variables after one year is because fatigue already has had its effect on these variables at baseline. Future research should aim to further explore the impact of fatigue on employment directly at the time of diagnosis. Finally, no significant associations were found with emotion-focused coping in the current study, while previous studies did find associations between coping and employment [4, 14]. It may be that we did not find significant associations because we combined the scores of two different questionnaires (UCL and CISS) and used the median as cutoff score for categorizing into low versus high emotion-focused coping style. Scores of the original questionnaires are not dichotomized and are therefore more sensitive to identify differences in emotion-focused coping. Future studies should include scores on the UCL or CISS to further investigate the association between emotion-focused coping and employment.

This study has several strengths. We were the first to include a healthy control group in studying employment in PwMS allowing us to make a distinction between generic and disease-specific predictors. Second, we only included recently diagnosed PwMS (≤ one year) which is rare in the literature so far, even though this period seems crucial for intervening when it comes to employment. Third, we included a relatively large number of variables representing three components from the ICF framework. Because we studied a combination of different factors from these three components, we were able to illustrate that not only different factors are at play when it comes to employment in recently diagnosed PwMS but also that the interplay between factors from these components might be of importance. Fourth, we used a Bayesian approach to estimate the effects, which allowed us to use the information provided by the healthy population as informative priors for the MS population and therefore increasing the precision and statistical power compared to a simple stratified analysis.

The current study has some limitations. Because data from two separate samples have been used, we were limited in choosing overlapping variables. We also had to make a selection of personal factors to ensure sufficient statistical power. Therefore, we were unable to include all possibly relevant environmental, physical, and psychosocial factors that may influence work difficulties and working hours in pwMS. In addition, not all data were complete. There were no available data yet on MSWDQ scores for healthy people after one year in one of the two datasets (the ongoing Temprano study). Also, the MSWDQ was developed for PwMS and connects with the difficulties PwMS are experiencing at work, thereby it is less suited for healthy people. It may be that healthy people are experiencing other difficulties at work which are not captured by using the MSWDQ. Future studies should aim to include a more comprehensive questionnaire of work difficulties so that comparisons can be made between healthy people and PwMS. Additionally, the findings from the current study cannot be generalized to all PwMS because only patients with a relapsing remitting disease course one year after diagnosis were included in both studies.

Concluding, a combination of baseline predictors (learning and memory, information processing speed, sex, age, conscientiousness, and the nature of the work tasks) that fit within three components of the ICF associate with perceived work difficulties and the number of working hours after one year. All associations, except for conscientiousness, were disease specific. Future studies aiming to improve employment in PwMS should consider early and multifaceted interventions focusing on training conscientiousness behavior, improving cognitive functioning, and adjusting job tasks where needed.

## Supplementary Information

Below is the link to the electronic supplementary material.

Supplementary file1 (DOCX 63 KB)

## Data Availability

Data used in this manuscript can be made available upon reasonable request from a qualified investigator.
