# Supplementary material for: What Predicts Changes in the Work Situation of Recently Diagnosed People with Multiple Sclerosis and do these Predictors also Apply to Healthy People?
Source: J Occup Rehabil. 2025 Feb 27;36(2):426–36. doi: 10.1007/s10926-025-10279-2 (PMC13099700; doi:10.1007/s10926-025-10279-2)
Supplement: Supplementary file 1 — Supplementary file1 (DOCX 63 KB) [file 10926_2025_10279_MOESM1_ESM.docx]

| Supplementary materials 1 Overview of the measures in the included studies: Temprano and MS@Work | |
| --- | --- |
| Temprano | **MS@Work** |
| Arm Function in Multiple Sclerosis Questionnaire – Short Form (AMSQ-SF) | Checklist Individual Strength-20-R* |
| Athens Insomnia Scale | Coping Inventory for Stressful  Situations |
| Checklist Individual Strength (CIS-20r)* | Caregiver Strain Index |
| Childhood Trauma Questionnaire-Short Form (CTQ-SF) | Empathy Quotient |
| Connor Davidson Resilience Scale (CD-RISC) | Every Day Problem Checklist |
| Health- and Lifestyle (including questions about work) | General questionnaire (Demographics, disease  characteristics, medication,  characteristics of current and  previous jobs, absenteeism,  presenteeism, work ability,  work-related problems and  accommodations and  important job aspects.) |
| Hospital Anxiety and Depression Scale (HADS-NL Dutch version)* | Hospital Anxiety and Depression  Scale* |
| Information about medication | Multiple Sclerosis Quality of Life-54 |
| List of Threatening Events Questionnaire (LTE) | Multiple Sclerosis Neuropsychological  Screening Questionnaire* |
| Multiple Sclerosis Neuropsychological Screening Questionnaire (MSNQ)* | Multiple Sclerosis Work Difficulties Questionnaire* |
| Multiple Sclerosis Work Difficulties Questionnaire (MSWDQ-23)* | Modified Fatigue Impact Scale-20 |
| Multiple Sclerosis Walking Scale | Neuropsychological test scores (MACFIMS)* |
| Neuropsychological test scores (MACFIMS)* | NEO Five-Factor Personality  Inventory* |
| NEO Five-Factor Inventory (NEO-FFI)* | Neuropsychiatric Inventory |
| Perceived Stress Scale (PSS) | Symptom Checklist-90-R  (sleep difficulties) |
| The multiple sclerosis impact scale (MSIS-29) | The Trail Making Test and subtests of the  Delis-Kaplan Executive Function System |
| Utrechtse Coping Lijst | Work Role Functioning  Questionnaire |

Note. In the MS@Work study, participants were measured at baseline, 6 months, 1 year, 2 years and 3 years after baseline. In the Temprano study participants were measured at baseline, after 12 months and after 24 months. * = overlapping measures between the two studies.

| Supplementary materials 2 Descriptive characteristics for participants in each complete-case analyses model | | | | |
| --- | --- | --- | --- | --- |
|  | Work difficulties after 1 year | | Working hours after 1 year | |
| Variable | **HC (n=97)**  **n(%)** | **MS (n=61)**  **n(%)** | **HC (n=129)**  **n(%)** | **MS (n=52)**  **n(%)** |
| Sex | 73(75.3%) female | 52(85.2%) female | 94(72.9%) female | 43(82.7%) female |
| Education |  |  |  |  |
| - Practical | 19(19.6%) | 21(34.4%) | 26(20.2%) | 19(36.5%) |
| - Theoretical | 78(80.4%) | 40(65.6%) | 103(79.8%) | 33(63.5%) |
| Disease modifying treatment |  |  |  |  |
| - None or first line | - | 53(86.9%) | - | 44(84.6%) |
| - Second line | - | 8(13.1%) | - | 8(15.4%) |
| Emotion-focused coping |  |  |  |  |
| - Low | 50(51.5%) | 39(63.9%) | 66(51.2%) | 33(63.5%) |
| - High | 47(48.5%) | 22(36.1%) | 63(48.8%) | 19(36.5%) |
| Work tasks |  |  |  |  |
| - Mentally burdensome | 76(78.4%) | 15(24.6%) | 89(69.0%) | 14(26.9%) |
| - Physically burdensome | 21(21.6%) | 46(75.4%) | 40(31.0%) | 38(73.1%) |
|  | **HC mean(SD)** | **MS mean(SD)** | **HC mean(SD)** | **MS mean(SD)** |
| Age in years | 41.06(10.62) | 37.15(8.85) | 40.05(10.96) | 36.31(8.87) |
| Fatigue | 22.39(10.23) | 33.02(11.74) | 22.95(10.62) | 31.83(11.73) |
| Information processing speed (z-score) | -0.06(0.99) | -0.22(1.01) | -0.01(0.96) | -0.22(1.09) |
| Learning and memory (z-score) | -0.02(0.53) | -0.14(0.73) | 0.01(0.57) | -0.14(0.77) |
| Conscientiousness | 48.08(5.29) | 46.43(4.45) | 47.50(5.73) | 46.13(4.66) |
| Work difficulties |  |  |  |  |
| - Baseline | 8.01(7.71) | 22.35(14.62) | 9.32(9.77) | 21.56(14.45) |
| - 1 year follow-up | 8.12(9.35) | 21.00(15.94) | - | - |
| Working hours per week |  |  |  |  |
| - Baseline | 35.37(8.40) | 28.84(10.34) | 34.52(9.11) | 29.10(10.14) |
| - 1 year follow-up | - | - | 33.87(9.09) | 28.50(10.48) |

*Note.* HC= healthy controls. MS= recently diagnosed people with multiple sclerosis. Education categories are combinations of Verhage[1] scores. Practical= scores 1-5, Theoretical= scores 6 and 7. Fatigue: range=8-56, higher=more fatigued. Information processing speed (z-score)**:** higher= better. Learning and memory (z-score**):** higher= better. Conscientiousness: range=27-60, higher=more conscientious. Work difficulties: higher= more work difficulties, baseline range= 0-67.05, 1 year follow-up range= 0-63.64. Working hours per week: baseline range= 0-70, 1 year follow-up range= 0-7

| Supplementary materials 3 Summary statistics work difficulties in healthy controls reported for each parameter directly from the Stan model output for the log transformed outcome | | | | | | | | | | | |
| --- | --- | --- | --- | --- | --- | --- | --- | --- | --- | --- | --- |
| Parameter | **mean** | **se_mean** | **sd** | **2.5%** | **25%** | **50%** | **75%** | **97.5%** | **n_eff** | **Rhat** | **95%CI** |
| alpha | 1.13 | 0.18 | 11.86 | -20.75 | -0.68 | 1.45 | 3.51 | 20.63 | 4511.73 | 1.00 | -20.75, 20.63 |
| alpha_source[1] | 0.04 | 0.37 | 18.77 | -20.83 | -1.44 | 0.00 | 1.46 | 22.07 | 2511.98 | 1.00 | -20.83, 22.07 |
| alpha_source[2] | 0.35 | 0.18 | 11.76 | -19.01 | -1.37 | 0.01 | 1.49 | 21.85 | 4499.45 | 1.00 | -19.01, 21.85 |
| phi | 1.19 | <0.00 | 0.24 | 0.79 | 1.02 | 1.17 | 1.34 | 1.74 | 25767.17 | 1.00 | 0.79, 1.74 |
| sigma_source | 7.06 | 0.38 | 20.43 | 0.15 | 1.06 | 2.48 | 5.85 | 44.09 | 2840.20 | 1.00 | 0.15, 44.09 |
| Sex (female vs. male) | 0.19 | <0.00 | 0.31 | -0.42 | -0.02 | 0.19 | 0.40 | 0.81 | 25279.69 | 1.00 | -0.42, 0.81 |
| Age | -0.01 | <0.00 | 0.01 | -0.03 | -0.01 | -0.01 | <0.00 | 0.02 | 22451.66 | 1.00 | -0.03, 0.02 |
| Education (theoretical vs practical) | -0.07 | <0.00 | 0.32 | -0.70 | -0.28 | -0.07 | 0.14 | 0.54 | 25606.15 | 1.00 | -0.70, 0.54 |
| Fatigue | 0.02 | <0.00 | 0.01 | -0.01 | 0.01 | 0.02 | 0.03 | 0.05 | 24380.86 | 1.00 | -0.01, 0.05 |
| Learning and memory | 0.35 | <0.00 | 0.24 | -0.13 | 0.18 | 0.35 | 0.51 | 0.83 | 27396.45 | 1.00 | -0.13, 0.83 |
| Information processing speed | -0.23 | <0.00 | 0.12 | -0.47 | -0.31 | -0.23 | -0.15 | 0.01 | 27975.29 | 1.00 | -0.47, 0.01 |
| Conscientiousness | -0.03 | <0.00 | 0.02 | -0.07 | -0.04 | -0.03 | -0.01 | 0.01 | 17895.32 | 1.00 | -0.07, 0.01 |
| Emotion focused coping (high vs low) | 0.18 | <0.00 | 0.25 | -0.31 | 0.02 | 0.18 | 0.35 | 0.66 | 25237.74 | 1.00 | -0.31, 0.66 |
| Work tasks (mentally burdensome vs physically) | 0.14 | <0.00 | 0.31 | -0.47 | -0.06 | 0.14 | 0.35 | 0.74 | 28651.61 | 1.00 | -0.47, 0.74 |
| Work difficulties baseline | 0.09 | <0.00 | 0.02 | 0.04 | 0.07 | 0.08 | 0.10 | 0.13 | 27453.47 | 1.00 | 0.04, 0.13 |
| Working hours baseline | 0.01 | <0.00 | 0.01 | -0.01 | 0.00 | 0.01 | 0.02 | 0.04 | 22418.31 | 1.00 | -0.01, 0.04 |

Note. Rhat is always very close to zero, showing good convergence and mixing of the posterior chains, and n_eff is always larger than 2000, except for one parameter when it is still larger than 600. This shows that we have enough independent posterior draws to obtain reliable summary statistics of the parameters (the recommendation is n_eff>100).

| Supplementary materials 4 Summary statistics working hours in healthy controls reported for each parameter directly from the Stan model output for the log transformed outcome | | | | | | | | | | | |
| --- | --- | --- | --- | --- | --- | --- | --- | --- | --- | --- | --- |
| Parameter | **mean** | **se_mean** | **sd** | **2.5%** | **25%** | **50%** | **75%** | **97.5%** | **n_eff** | **Rhat** | **95%CI** |
| alpha | 2.38 | 0.02 | 1.25 | 0.33 | 2.13 | 2.41 | 2.66 | 4.32 | 2904.91 | 1.00 | 0.33, 4.32 |
| alpha_source[1] | 0.05 | 0.02 | 1.23 | -1.86 | -0.10 | 0.01 | 0.14 | 2.11 | 2874.39 | 1.00 | -1.86, 2.11 |
| alpha_source[2] | 0.01 | 0.02 | 1.23 | -1.92 | -0.14 | -0.01 | 0.09 | 2.06 | 2877.27 | 1.00 | 1.92, 2.06 |
| phi | 72.54 | 0.12 | 19.21 | 42.20 | 59.11 | 69.65 | 83.40 | 117.30 | 25477.15 | 1.00 | 42.20, 117.30 |
| sigma_source | 0.83 | 0.03 | 1.80 | 0.01 | 0.09 | 0.29 | 0.89 | 4.75 | 4014.26 | 1.00 | 0.01, 4.75 |
| Sex (female vs. male) | 0.00 | <0.00 | 0.05 | -0.09 | -0.03 | 0.00 | 0.04 | 0.10 | 24466.07 | 1.00 | -0.09, 0.10 |
| Age | -0.00 | <0.00 | <0.00 | -0.01 | -0.00 | -0.00 | -0.00 | 0.00 | 2563.58 | 1.00 | -0.01, 0.00 |
| Education (theoretical vs practical) | -0.05 | <0.00 | 0.05 | -0.15 | -0.08 | -0.05 | -0.01 | 0.06 | 25617.77 | 1.00 | -0.15, 0.06 |
| Fatigue | <-0.00 | <0.00 | <0.00 | -0.01 | -0.00 | -0.00 | -0.00 | 0.00 | 24871.89 | 1.00 | -0.01, 0.00 |
| Learning and memory | <0.00 | <0.00 | 0.04 | -0.07 | -0.02 | 0.00 | 0.03 | 0.08 | 2402.02 | 1.00 | -0.07, 0.08] |
| Information processing speed | -0.03 | <0.00 | 0.02 | -0.07 | -0.04 | -0.03 | -0.01 | 0.01 | 5222.90 | 1.00 | -0.07, 0.01 |
| Conscientiousness | 0.01 | <0.00 | <0.00 | <0.00 | 0.01 | 0.01 | 0.01 | 0.02 | 9237.29 | 1.00 | 0.00, 0.02 |
| Emotion focused coping (high vs low) | -0.01 | <0.00 | 0.04 | -0.09 | -0.04 | -0.01 | 0.02 | 0.08 | 3815.59 | 1.00 | -0.09, 0.08 |
| Work tasks (mentally burdensome vs physically) | 0.06 | <0.00 | 0.05 | -0.04 | 0.03 | 0.06 | 0.10 | 0.16 | 1027.84 | 1.01 | -0.04, 0.16 |
| Work difficulties baseline | <0.00 | <0.00 | <0.00 | <-0.00 | -0.00 | 0.00 | 0.00 | 0.01 | 677.84 | 1.01 | -0.00, 0.01 |
| Working hours baseline | 0.02 | <0.00 | <0.00 | 0.02 | 0.02 | 0.02 | 0.03 | 0.03 | 21715.42 | 1.00 | 0.02, 0.03 |

Note. Rhat is always very close to zero, showing good convergence and mixing of the posterior chains, and n_eff is always larger than 2000, except for one parameter when it is still larger than 600. This shows that we have enough independent posterior draws to obtain reliable summary statistics of the parameters (the recommendation is n_eff>100).

| Supplementary materials 5 Summary statistics work difficulties in people with MS reported for each parameter directly from the Stan model output for the log transformed outcome | | | | | | | | | | | |
| --- | --- | --- | --- | --- | --- | --- | --- | --- | --- | --- | --- |
| Parameter | **mean** | **se_mean** | **sd** | **2.5%** | **25%** | **50%** | **75%** | **97.5%** | **n_eff** | **Rhat** | **95%CI** |
| alpha | 2.12 | 0.03 | 1.92 | -0.72 | 1.43 | 2.06 | 2.71 | 5.26 | 3808.43 | 1.00 | -0.72, 5.26 |
| alpha_source[1] | 0.03 | 0.03 | 1.78 | -2.91 | -0.17 | 0.05 | 0.35 | 2.71 | 3442.35 | 1.00 | -2.91, 2.71 |
| alpha_source[2] | -0.16 | 0.03 | 1.78 | -3.15 | -0.37 | -0.05 | 0.16 | 2.49 | 3442.13 | 1.00 | -3.15, 2.49 |
| phi | 2.48 | 0.01 | 0.60 | 1.50 | 2.06 | 2.42 | 2.84 | 3.85 | 5008.24 | 1.00 | 1.50, 3.85 |
| sigma_source | 1.28 | 0.04 | 2.51 | 0.04 | 0.24 | 0.60 | 1.41 | 6.51 | 3136.53 | 1.00 | 0.04, 6.51 |
| Sex (female vs. male) | -0.11 | 0.00 | 0.29 | -0.70 | -0.30 | -0.10 | 0.08 | 0.44 | 27915.57 | 1.00 | -0.70, 0.44 |
| Age | 0.01 | 0.00 | 0.01 | -0.02 | -0.00 | 0.01 | 0.02 | 0.03 | 23872.67 | 1.00 | -0.02, 0.03 |
| Education (theoretical vs practical) | 0.32 | 0.00 | 0.20 | -0.07 | 0.18 | 0.32 | 0.45 | 0.72 | 24200.05 | 1.00 | -0.07, 0.72 |
| Fatigue | 0.01 | 0.00 | 0.01 | -0.01 | 0.00 | 0.01 | 0.02 | 0.03 | 12087.89 | 1.00 | -0.01, 0.03 |
| Learning and memory | 0.02 | 0.00 | 0.11 | -0.20 | -0.06 | 0.02 | 0.09 | 0.23 | 26171.63 | 1.00 | -0.20, 0.23 |
| Information processing speed | -0.20 | 0.00 | 0.09 | -0.37 | -0.25 | -0.19 | -0.14 | -0.03 | 25815.27 | 1.00 | -0.37, -0.03 |
| Conscientiousness | -0.03 | 0.00 | 0.02 | -0.06 | -0.04 | -0.03 | -0.02 | 0.00 | 20833.64 | 1.00 | -0.06, 0.00 |
| Emotion focused coping (high vs low) | 0.08 | 0.00 | 0.17 | -0.25 | -0.03 | 0.08 | 0.19 | 0.41 | 26283.48 | 1.00 | -0.25, 0.41 |
| Work tasks (mentally burdensome vs physically) | 0.18 | 0.00 | 0.21 | -0.23 | 0.04 | 0.18 | 0.32 | 0.60 | 26116.45 | 1.00 | -0.23, 0.60 |
| Work difficulties baseline | 0.04 | 0.00 | 0.01 | 0.03 | 0.04 | 0.04 | 0.05 | 0.06 | 23252.90 | 1.00 | 0.03, 0.06 |
| Working hours baseline | 0.01 | 0.00 | 0.01 | -0.00 | 0.01 | 0.01 | 0.02 | 0.03 | 28031.11 | 1.00 | -0.00, 0.03 |
| Disease modifying treatment (2^nd^ line vs 1^st^ line) | -0.14 | 0.00 | 0.33 | -0.77 | -0.36 | -0.15 | 0.07 | 0.51 | 25901.34 | 1.00 | -0.77, 0.51 |

Note. Rhat is always very close to zero, showing good convergence and mixing of the posterior chains, and n_eff is always larger than 2000, except for one parameter when it is still larger than 600. This shows that we have enough independent posterior draws to obtain reliable summary statistics of the parameters (the recommendation is n_eff>100).

| Supplementary materials 6 Summary statistics working hours in people with MS reported for each parameter directly from the Stan model output for the log transformed outcome | | | | | | | | | | | |
| --- | --- | --- | --- | --- | --- | --- | --- | --- | --- | --- | --- |
| Parameter | **mean** | **se_mean** | **sd** | **2.5%** | **25%** | **50%** | **75%** | **97.5%** | **n_eff** | **Rhat** | **95%CI** |
| alpha | 2.16 | 0.04 | 2.36 | -0.73 | 1.85 | 2.17 | 2.47 | 4.96 | 2869.25 | 1.00 | -0.73, 4.96 |
| alpha_source[1] | 0.13 | 0.04 | 2.36 | -2.62 | -0.14 | 0.10 | 0.39 | 3.00 | 2858.13 | 1.00 | -2.62, 3.00 |
| alpha_source[2] | -0.10 | 0.04 | 2.36 | -2.87 | -0.38 | -0.09 | 0.15 | 2.75 | 2859.30 | 1.00 | -2.87, 2.75 |
| phi | 12.26 | 0.03 | 4.81 | 5.80 | 8.91 | 11.34 | 14.60 | 24.04 | 25059.80 | 1.00 | 5.80, 24.04 |
| sigma_source | 1.38 | 0.07 | 3.78 | 0.06 | 0.27 | 0.63 | 1.45 | 6.71 | 2728.15 | 1.00 | 0.06, 6.71 |
| Sex (female vs. male) | -0.29 | <0.00 | 0.13 | -0.55 | -0.37 | -0.29 | -0.20 | -0.05 | 26394.76 | 1.00 | -0.55, -0.05 |
| Age | -0.01 | <0.00 | 0.00 | -0.02 | -0.01 | -0.01 | -0.01 | <-0.00 | 27092.53 | 1.00 | -0.02, -0.00 |
| Education (theoretical vs practical) | 0.10 | <0.00 | 0.07 | -0.03 | 0.05 | 0.10 | 0.15 | 0.24 | 26813.99 | 1.00 | -0.03, 0.24 |
| Fatigue | <0.00 | <0.00 | 0.00 | <-0.00 | <0.00 | <0.00 | <0.00 | 0.01 | 27935.29 | 1.00 | -0.00, 0.01 |
| Learning and memory | 0.08 | <0.00 | 0.03 | 0.03 | 0.07 | 0.08 | 0.10 | 0.14 | 28076.30 | 1.00 | 0.03, 0.14 |
| Information processing speed | 0.08 | <0.00 | 0.02 | 0.04 | 0.06 | 0.08 | 0.09 | 0.12 | 28594.39 | 1.00 | 0.04, 0.12 |
| Conscientiousness | 0.02 | <0.00 | 0.00 | 0.01 | 0.02 | 0.02 | 0.02 | 0.03 | 27340.19 | 1.00 | 0.01, 0.03 |
| Emotion focused coping (high vs low) | -0.03 | <0.00 | 0.04 | -0.11 | -0.05 | -0.03 | <0.00 | 0.06 | 27602.53 | 1.00 | -0.11, 0.06 |
| Work tasks (mentally burdensome vs physically) | 0.31 | <0.00 | 0.06 | 0.20 | 0.27 | 0.31 | 0.35 | 0.42 | 25384.60 | 1.00 | 0.20, 0.42 |
| Work difficulties baseline | <-0.00 | <0.00 | 0.00 | -0.01 | <-0.00 | <-0.00 | <-0.00 | <0.00 | 28338.33 | 1.00 | -0.01, 0.00 |
| Working hours baseline | 0.02 | <0.00 | 0.00 | 0.02 | 0.02 | 0.02 | 0.02 | 0.03 | 27959.27 | 1.00 | 0.02, 0.03 |
| Disease modifying treatment (2^nd^ line vs 1^st^ line) | 0.08 | <0.00 | 0.17 | -0.25 | -0.03 | 0.07 | 0.19 | 0.41 | 27706.06 | 1.00 | -0.25, 0.41 |

Note. Rhat is always very close to zero, showing good convergence and mixing of the posterior chains, and n_eff is always larger than 2000, except for one parameter when it is still larger than 600. This shows that we have enough independent posterior draws to obtain reliable summary statistics of the parameters (the recommendation is n_eff>100).

**References**

1. Verhage F. Intelligentie en leeftijd; onderzoek bij Nederlanders van twaalf tot zevenenzeventig jaar. Assen: Van Gorcum; 1964.
